# The therapist’s role in the implementation of internet-based cognitive behavioural therapy for patients with depression: study protocol

**DOI:** 10.1186/s12888-016-1045-9

**Published:** 2016-09-30

**Authors:** Mayke Mol, Els Dozeman, Digna J. F. van Schaik, Christiaan P. C. D. Vis, Heleen Riper, Jan H Smit

**Affiliations:** 1 Department of Research & Innovation, GGZ inGeest, A.J. Ernststraat 1187, 1081 HL Amsterdam, The Netherlands; 2 EMGO Institute for Health Care and Research and Department of Psychiatry, VU University Medical Centre, Van der Boechorststraat 1, 1081 BT Amsterdam, The Netherlands; 3 Department of Clinical Psychology, VU University Amsterdam, Van der Boechorststraat 1, 1081 BT Amsterdam, The Netherlands

**Keywords:** Implementation, Therapist’s role, E-mental health, Routine practice, Depression, Internet-based cognitive behavioural therapy, Online treatment, Blended treatment

## Abstract

**Background:**

Internet-based Cognitive Behavioural Therapy (iCBT) for the treatment of depressive disorders is innovative and promising. Various studies have demonstrated large effect sizes up to 2.27, but implementation in routine practice lags behind. Mental health therapists play a significant role in the uptake of internet-based interventions. Therefore, it is interesting to study factors that influence the therapists in whether they apply internet-based therapy or not. This study, as part of the European implementation project MasterMind, aims to identity the factors that promote or hinder therapists in the use of iCBT in depression care.

**Methods/Design:**

The uptake of iCBT by therapists in routine mental health care practice for the treatment of depression will be evaluated by a mixed method approach, to provide an understanding of the implementation factors (quantitative), and to ascertain the facilitating and hindering factors in the involvement of therapists in the implementation of iCBT (qualitative). The involvement of therapists in the implementation of iCBT is analysed following the RE-AIM framework on the five dimensions Reach, Efficacy/Effectiveness, Adoption, Implementation, and Maintenance. This enables us to evaluate the reach of therapists, the impact of iCBT on depression care, the extent to which therapists adopt iCBT, the extent to which iCBT is delivered as intended, and how iCBT can be maintained over time.

**Discussion:**

The results will provide valuable insight into the role of therapists in the implementation of iCBT for depression in secondary mental health care settings. They will result in concrete recommendations for how therapists can be facilitated in implementing and up-scaling iCBT for depression.

## Background

Despite its potential advantages and the evidence for its effectiveness, the implementation of internet-based Cognitive Behavioural Therapy (iCBT) for depression [1] in routine mental health care is lower than expected. Since 2000 more than 100 trials on iCBT have been conducted for various clinical disorders, such as depression, anxiety and chronic pain [2]. Most of the included studies focussed on clinical efficacy, and a small minority on cost effectiveness. The results from these various studies are promising, with large within-group effect sizes in the treatment of depression ranging from 0.38 to 2.27, with a mean of 0.94. Treatment with iCBT showed equivalent effects compared to conventional CBT. However, the use of iCBT is not scaled up to the extent that it actually helps to reduce the disease burden of depression. In the yearly national report on eHealth in the Netherlands only 6 % of a representative sample of mental health patients used online treatment in combination with usual care in 2015 [3]. The low uptake of iCBT is recognised by multiple health care organisations and policy makers across different countries but is not yet well understood.

The MasterMind project (Management of mental health disorders through advanced technology and services-telehealth for the MIND) [4] aims to provide understanding of the factors that promote or hinder the uptake of iCBT for depression throughout Europe from a broad perspective. MasterMind explores the role of different stakeholders that are involved in the successful implementation of the intervention (e.g. patients, therapists, mental healthcare organisations, regional mental healthcare systems) [5]. In Mastermind videoconferencing services will also be implemented in routine mental healthcare. In the current study protocol we focus specifically on the role of therapists in implementing iCBT in routine practice in the Netherlands.

To evaluate the role of the therapists in interaction with other barriers and facilitators at multiple levels of mental health care, the use of a framework and mixed method approach is needed [6, 7]. In this study the RE-AIM framework [8, 9] is used to structure the evaluation of the different implementation factors [10]. RE-AIM is widely used in implementation research, especially in e-mental health research. RE-AIM stands for: Reach, Efficacy or Effectiveness, Adoption, Implementation, and Maintenance. Together these dimensions help shed light on the barriers and facilitators and can ease the transition from research to practice. The RE-AIM framework is applied for evaluations at an individual level and an organizational level, as each level can provide valuable independent information on intervention impact. Reach and Effectiveness have individual levels of impact, whereas Adoption and Implementation have organisational levels of impact. Maintenance can have both an individual and an organisational level of impact [11].

The current situation in the Netherlands is that most mental health care organisations have adopted iCBT; there are many different providers for online platforms to choose from and almost every mental health care organisation already has on online platform in use for the internet-based treatment of mental disorders. However, mental health care organisations have different developmental levels regarding the implementation of iCBT in their care system. Some organisations have worked with online interventions for several years now, while others have just started implementing iCBT. Although many therapists in the mental health care organisations have been trained in using an online platform, a recent naturalistic study on the implementation of iCBT in a mental health service organisation in the Netherlands showed that only 3.6 % of the patients with depression or anxiety were offered iCBT (in a blended format: face-to-face and online sessions) by 18 % of the trained therapists over a time period of 3 years [12]. Thus, the reach of the intervention is still limited. A similar result was found by Carper and others [13] who showed that in the US there is a lack of awareness of iCBT in depression care among patients and clinicians. The findings concluded that a more widespread dissemination of iCBT interventions is needed.

In the literature some attention has already been paid to therapist related factors that play a role in the implementation of iCBT. Donovan et al. [14] found that knowledge of iCBT among mental health care professionals can play a role in the uptake of iCBT: greater knowledge of the intervention’s effectiveness was associated with fewer perceived disadvantages of iCBT in general, and more circumstances under which iCBT was perceived to be useful. Wilhemsen and others [15] indicated that lack of time, varied practice, inadequate knowledge of the program and changing professional’s habits are hindering factors in the implementation of iCBT. A qualitative study conducted by Kivi et al. [16] with four therapists identified similar barriers to implementing iCBT in mental health care. Therapists perceived a lack of knowledge, skills, resources and time and they had the feeling that they never fully mastered the protocol. Another explorative qualitative study with 11 therapists showed that several therapists see “iCBT as focused, structured, evidence-based, clear and safe” [17]. Although they feel that iCBT in general can provide the therapist more work-time control because of the structured nature, several therapists view iCBT as inferior to face-to-face CBT and think that a working alliance in the latter is achieved faster and more easily.

It is important to take clinical implications and therapists’ concerns into account when implementing iCBT in routine practice. These considerations may provide more insight in how to optimise the uptake by therapists. In this study we will further explore factors regarding the therapists’ role in implementing iCBT for depression in adults in secondary mental health care

### Study objectives

There is a need for more information about the barriers therapists experience in adopting and implementing iCBT and how to overcome these barriers. To this end we focus on the needs of the therapist and (patient and organisation-related) facilitating and impeding factors. The MasterMind study provides an excellent opportunity to study these therapist factors. The objectives of this study are:

1. To gain insight into the extent to which therapists integrate iCBT in their routine practice.
2. To increase insight into the factors that promote or hinder the use of iCBT by therapists in depression care.
3. To provide recommendations on how to overcome barriers for therapists in the implementation of iCBT in routine depression care.

In order to obtain insight into strategies for optimising therapist uptake of iCBT we use the RE-AIM framework to answer the following questions:

- Reach: What is the proportion and representativeness of therapists in the participating mental health organisations that offer iCBT for depression during the study?
- Effectiveness: What is the (positive and negative) impact of iCBT on the therapists regarding perceived effectiveness, satisfaction and usability? How is this related to patient outcomes regarding symptom reduction, satisfaction and usability?
- Adoption: To what extent do mental health care organisations adopt iCBT; how is the therapist facilitated in this and which efforts are made to this end in the participating mental health organisations?
- Implementation: To what extent is iCBT implemented as intended in routine practice? What are implementation barriers and facilitators from therapists’ perspective?
- Maintenance: To what extent does iCBT become a sustained part of routine practice, and what are facilitating and hindering factors in maintenance?

## Methods and design

### Study design

To reach the study objectives, the uptake of iCBT for depression in several mental health care practices in the Netherlands will be monitored. This multicentre evaluation will assess the involvement of therapists in the implementation of iCBT in interaction with organisational and patient factors. The study will investigate how implementation is reflected in the extent to which organisations adopt the intervention, and how the therapist’s perspective is affected by patient related factors such as changes in symptoms, satisfaction and perceived usability of the iCBT. A mixed-method approach will be used in order to provide an understanding of the implementation factors (quantitative) and to evaluate the facilitating and hindering factors in the involvement of therapists in the implementation of iCBT (qualitative) [8, 18, 19].

### Sample

For the MasterMind study on an European level, the target number of 5230 participants is based on purposive quota sampling meaning the number of participants needed to be enrolled to regard the interventions as being implemented in routine practice after the study is finished, for the study in the Netherlands the target number is 300 participants and their therapists [5]. With estimating the sample size, feasibility and service delivery characteristics of iCBT in routine care in the Netherlands are taken into account. Ten mental health care organisations spread throughout the Netherlands will be approached. The selection of organisations is based on purposive sampling, meaning the organisations have different stages of iCBT implementation, as measured by the number of trained therapists and patients treated with iCBT; the organisations are located in different parts of the Netherlands and may use different iCBT treatment platforms for depression in secondary mental health care. The diversity of the participating organisations is necessary to enrich the (qualitative) data needed for the evaluation of the therapists’ involvement in the Netherlands. In the participating organisations we will approach key stakeholders at different levels such as eHealth project managers and directors.

### Therapists

The therapists who are trained in iCBT will be invited to participate in the study. The therapists can be psychologists, mental health nurses or psychiatrists. Some of them are already experienced in delivering iCBT for depression; others are trained but not yet experienced.

### Patients

#### Inclusion criteria

Patients from the participating mental health care organisations will be invited for participation if they are aged 18 years or older, have a mild, moderate or severe depression as a primary diagnosis according to the clinician, and are indicated for cognitive behavioural treatment for depression following routine care procedures. All patients need to explicitly consent to taking part in the study. Patients may provide electronic consent to use their data for the evaluation study.

#### Exclusion criteria

Patients will be excluded from the study if they a) do not have a valid email address and do not have a computer with internet access b) do not have adequate Dutch language skills; verbal and written.

### Representatives of the mental healthcare organisations

From each mental healthcare organisation representatives will be invited to take part in the study.

The representatives are persons who have a substantive decision-making position in the implementation of iCBT in the organisation’s mental healthcare practice.

### Intervention

Cognitive behavioural therapy relies on cognitive input and behavioural change and is well structured. This makes it suitable for an innovative information-technology based application [20]. In such an application an online treatment platform provides patient and therapist with an overview of the treatment sessions, information and exercises. Through the platform the patient can access psycho-education, treatment information and exercises at any time within the home environment. Furthermore, the therapist can monitor the progress of the patient online. With the integration of technology, iCBT can have several promising advantages; iCBT may improve access to treatment, because it can lower the barriers to seeking mental health care; iCBT can promote self-monitoring and self-management of the patient; iCBT might reduce treatment costs because of a reduction in face-to-face time [21]; the online platform is available for the patient 24 h, 7 days a week; and the online sessions can be repeated [22]. Furthermore, iCBT may improve the quality of delivery of the treatment, because of the standardised nature that gives a clear overview of the treatment, for both therapist and patient.

Most mental health care organisations in the Netherlands have contracts with providers of web-based treatments for common mental disorders and are therefore able to offer iCBT for depression. The organisations use various secure web-based online treatment platforms. In routine secondary mental health care, iCBT is mostly delivered in a blended format, consisting of the combination of online treatment modules and face-to-face sessions. The iCBT treatment modules are based on evidence based treatment protocols for face-to-face cognitive behavioural therapy and are in agreement with the Dutch multidisciplinary guidelines for depression [23]. The core components of the treatment are: 1) psycho-education, 2) cognitive restructuring, 3) behavioural activation, and 4) relapse prevention. As there is a distinction in secondary healthcare in the Netherlands between basic mental health care (mild to moderate depression), and specialised mental health care (more complex moderate to severe depression) the number of iCBT sessions differs between these health care levels: up to a maximum of 10–15 sessions in basic mental health care and usually 15–20 sessions in specialised mental health care. At both health care levels, in blended treatment, the number of face-to-face sessions is reduced because half or more of the face-to-face sessions are replaced by online treatment sessions [24, 25].

### Outcome measures

The outcomes will be structured according to the RE-AIM framework. The concepts of the multidisciplinary assessment outcomes will be used to obtain a better understanding of the change in mental health care delivery and to help make decisions about the implementation more explicit and transparent. In this study the RE-AIM framework was somewhat adapted to fit to our focus on the therapists’ role, as was done earlier by Boersma et al. [26, 27]. *Reach* is the proportion and representativeness of therapists in the participating mental health organisations that offer iCBT for depression. *Effectiveness* is the impact of iCBT on therapists regarding perceived effectiveness, satisfaction and usability of iCBT and the impact of iCBT on patients regarding symptom reduction, satisfaction and usability. *Adoption* is the extent to which mental health care organisations adopt iCBT and how the therapist is facilitated in this. *Implementation* is the extent to which iCBT is implemented as intended in routine practice, including implementation barriers and facilitators from the therapist’s perspective. *Maintenance* is the extent to which iCBT becomes part of the normal routine of therapists. In other words, to have an impact on depression care, iCBT must be adopted by the mental health care organisations and therapists, reach the therapists who deliver the intervention, be implemented as intended, effectively improve outcomes and be maintained over time. The definitions of the RE-AIM frame work are presented in Table 1. In Table 2 an overview is given of the RE-AIM dimensions, the related content, factors and measures.

**Table 1:** Definitions of the five RE-AIM dimensions and the definitions in this study

Dimension RE-AIM (level)	Definition RE-AIM (Glasgow et al. 1999)	Definition RE-AIM in this study
Reach (therapist)	The absolute number, proportion, and representativeness of individuals who are willing to participate in a given initiative, intervention, or program.	The number, proportion and representativeness of therapists in the participating mental health organisations that offered iCBT for depression during the study.
Effectiveness (patient and therapist)	The impact of an intervention on important outcomes, including potential negative effects, quality of life, and economic outcomes.	The (positive and negative) impact of iCBT on the therapists and patients regarding perceived effectiveness, satisfaction and usability
Adoption (organisation)	The absolute number, proportion, and representativeness of settings and intervention agents (people who deliver the program) who are willing to initiate a program.	The extent to which mental health care organisations adopt iCBT and how the therapist is facilitated in this.
Implementation (therapist and organisation)	At the setting level, implementation refers to the intervention agents’ fidelity to the various elements of an intervention’s protocol, including consistency of delivery as intended and the time and cost of the intervention.	The extent to which iCBT is implemented as intended in routine practice, including implementation barriers and facilitators from the therapist’s perspective.
Maintenance (therapist and organisation)	The extent to which a program becomes institutionalized or part of standard organizational practices and policies.	The extent to which iCBT becomes a sustained part of standard practice and facilitating and hindering factors from therapists’ and organisational perspective.

**Table 2:** Overview of the RE-AIM dimensions, the related content and measures

RE-AIM dimension	Content			Measures/factors
Adoption	Participating Organisations: *n* and %			Characteristics of adopters’ Influencing factors: region, size, capacity, type, previous experience with other platforms
Reach	Total potential therapists, *n* ↓			
	Therapists eligible *n* and % ↓	Therapists excluded *n*, % and reasons		
	Therapists enrolled *n* and %	Therapists who decline *n*, % and reasons	Therapists not contacted/other *n* and %	Characteristics of enrollers vs decliners. Influencing factors: mandatory vs voluntary, available resources.
Implementation	Extent iCBT is delivered by therapists (as in protocol)			Extent iCBT is delivered as intended. Influencing factors: complexity of the intervention, costs in time and money, training, implementation activities, adaptations
Effectiveness	Impact of ICBT on therapists regarding perceived effectiveness, satisfaction and usability			The positive and negative impact of ICBT on therapists regarding perceived effectiveness, satisfaction and usability and related patient outcomes. Influencing factors: evidence for effectiveness of iCBT, impact across subgroups
Maintenance	Extent organisations maintain and/or modify iCBT			The extent iCBT becomes a sustained part of routine practice. Influencing factors: benefits vs costs. Amount of training, technical assistance, funding

### Measurements

Qualitative and quantitative measures will be used to help understand the factors related to the implementation of iCBT according to the RE-AIM framework. Focus-group interviews and semi-structured interviews with the therapists will be conducted face-to-face at the end of the study (after 24 months of data collection). The interviews with therapists will focus on the experiences and perceptions of the trained therapists who participated in delivering iCBT in their daily work. In addition, interviews will be held with trained therapists who do not deliver the treatment, in order to explore their reasons and their perceptions of iCBT. In the focus groups with the therapists discussion on the following themes will be held: the patient (needs, profile, safety), the therapist (needs and profile), implementation barriers and facilitators and usability and satisfaction with iCBT. Prior to the focus-group interviews and semi-structured interviews, participants will be asked to fill out a short questionnaire to obtain general information about the interviewees and to prepare them for the interviews. If therapists stop working with iCBT (due to other jobs, positions), they will be asked for their input at an earlier time point.

The semi-structured interviews with key figures of the participating mental health care organisations will be conducted face-to-face and they will also take place at the end of study. In the interviews the focus will be on implementation barriers and facilitators, usability, satisfaction and maintenance, from an organisational perspective.

Interviewers and moderators for the focus groups will be provided with training and comprehensive interview guides including tips for interview techniques as well as suitable questions. In addition, relevant documents provided by the mental health care organisations will be reviewed (e.g. implementation and quality improvement plans, treatment protocols).

Quantitative data will be collected at baseline and at the end of treatment from patients, and from therapists at the end of the study. Information on demographic characteristics of the therapists that will be collected comprises age, gender, education, profession, years of professional experience, number of hours working and experience with iCBT. From patients demographic characteristics will be collected about age, gender, education, employment, marital status and use of antidepressant medication. In addition, to measure symptoms of depression, we will adhere to routine practice and this will be registered in terms of several symptom questionnaires, depending on the questionnaires used at the participating organisation. Health care organisations in the Netherlands use Routine Outcome Measurements (ROM). ROM consists of a broad selection of validated instruments to monitor treatment effects. The use of ROM is a mandatory component of treatment as required by health insurance companies. The most frequently used questionnaires are the Patient Health Questionnaire (PHQ-9) [28], the Inventory of Depressive Symptoms (IDS-SR) [29, 30] and the Quick Inventory of Depressive Symptomatology (QIDS) [31].

Perceived usability and satisfaction with iCBT will be measured from both therapists’ and patients’ perspective. Patient treatment satisfaction will be measured with the 8 item Client Satisfaction Questionnaire (CSQ-8) [32]. System usability will be measured with the System Usability Scale (SUS) [33]. The perceived satisfaction and usability of iCBT by patients will be measured at the end of treatment. In addition, therapist satisfaction and usability of iCBT will be measured at the end of the study; satisfaction will be measured with the 3 items CSQ and usability will be measured with the SUS.

To assess the extent to which the treatment is delivered by protocol, the usage data from the online platforms will be gathered (e.g. number of online sessions and usage of other platform functionalities).

### Statistical analyses

Analysis will be of a summative nature combining quantitative data with qualitative data [34, 35] to draw a broad picture of implementation and to describe the role of the therapist in the uptake of iCBT in connection with the patient and the organisation. On a patient level, data of all participants will be included in the statistical analysis when they are eligible and agree to receiving treatment, regardless whether the participants complete their treatment or not.

The evaluation of the implementation will be performed by using the outcomes as described above following the RE-AIM dimensions. Descriptive analyses (frequencies, means and percentages) of the quantitative data (demographics of patients and therapists, depressive symptoms, perceived satisfaction and perceived usability) will be performed with the Statistical Package for the Social Sciences (SPSS), version 22.0. The qualitative data collected during the focus groups (including field notes) and the semi-structured interviews will be audiotaped and transcribed verbatim. We will use standard thematic content analysis techniques by the following steps: 1) familiarising and summarising the data 2) identifying codes and themes 3) coding the data and 4) organising the codes and themes [36]. In order to ensure reliability, agreement on codes and concepts between the members of the research team will be sought. Qualitative data analyses will be performed by using the software Atlas-ti.7.

## Discussion

The primary aim of the study is to gain insight into the extent to which therapists integrate internet-based Cognitive Behavioural Therapy (iCBT) in routine depression care in the Netherlands. The second aim is to gather insight into the factors that promote or hinder the implementation of iCBT. More specifically, we want to gain knowledge on the role of the therapist in the implementation of iCBT, in relation to patient and organisational factors; how do therapists evaluate iCBT, what resources are available and are needed, and what decision-making processes are important? The third aim is to provide recommendations for implementing iCBT in different contexts and organisations, in order to make high quality treatment more widely available for adults with depression.

The strength of this protocol is its multifaceted evaluation with a focus on the role of the therapist in relation to the organisation and the patients with depression. A second key strength is the mixed method approach to incorporate quantitative and qualitative aspects of the implementation outcomes. Glasgow [8] argues that qualitative data can help in interpreting quantitative results, “such as why potential users decline participation or why they do not remain engaged over time” and that “qualitative measures can be very helpful in understanding contextual issues”. The focus groups and interviews with therapists will give us more insight into how to implement iCBT in routine practice. Furthermore, the semi-structured interviews with representatives of the mental health care organisations will contribute to a better understanding of the implementation on an organisational level. The use of multiple data sources will provide a detailed understanding of how iCBT can be implemented. The RE-AIM framework helps to guide data collection and analysis for the evaluation. To our knowledge, this is one of the first studies to examine the implementation of iCBT for depression in routine mental health care systematically.

In sum, the findings of this study will provide a number of implications for future practice. When we know more about the barriers and facilitators for up-scaling and maintaining interventions and have more knowledge about the effective strategies that will help identify and characterise these, it will help us make mental health care for depression more widely available and effective in future practice.

## Abbreviations

CSQ: Client satisfaction questionnaire
EPD: Electronic patient dossier
iCBT: Internet-based cognitive behavioural therapy
IDS-SR: Inventory of depressive symptoms
PHQ: Patient health questionnaire
RE-AIM: Reach, effectiveness, adoption, implementation, maintenance
ROM: Routine outcome measurement
SUS: System usability scale
